# Phenotypic Analysis of Fourier-Transform Infrared Milk Spectra in Dairy Goats

**DOI:** 10.3390/foods12040807

**Published:** 2023-02-14

**Authors:** Bartolo de Jesús Villar-Hernández, Nicolò Amalfitano, Alessio Cecchinato, Michele Pazzola, Giuseppe Massimo Vacca, Giovanni Bittante

**Affiliations:** 1 Departamento de Zootecnia, Universidad Autónoma Chapingo, Texcoco 56230, Mexico; 2 Department of Agronomy, Food and Natural Resources, Animals and Environment (DAFNAE), University of Padova, Viale dell’Università 16, 35020 Legnaro, Italy; 3 Department of Veterinary Medicine, University of Sassari, 07100 Sassari, Italy

**Keywords:** FTIR, mid-infrared, caprine milk, milk absorbance spectra, variance components, sources of variation

## Abstract

The infrared spectrum of bovine milk is used to predict many interesting traits, whereas there have been few studies on goat milk in this regard. The objective of this study was to characterize the major sources of variation in the absorbance of the infrared spectrum in caprine milk samples. A total of 657 goats belonging to 6 breeds and reared on 20 farms under traditional and modern dairy systems were milk-sampled once. Fourier-transform infrared (FTIR) spectra were taken (2 replicates per sample, 1314 spectra), and each spectrum contained absorbance values at 1060 different wavenumbers (5000 to 930 × cm^−1^), which were treated as a response variable and analyzed one at a time (i.e., 1060 runs). A mixed model, including the random effects of sample/goat, breed, flock, parity, stage of lactation, and the residual, was used. The pattern and variability of the FTIR spectrum of caprine milk was similar to those of bovine milk. The major sources of variation in the entire spectrum were as follows: sample/goat (33% of the total variance); flock (21%); breed (15%); lactation stage (11%); parity (9%); and the residual unexplained variation (10%). The entire spectrum was segmented into five relatively homogeneous regions. Two of them exhibited very large variations, especially the residual variation. These regions are known to be affected by the absorbance of water, although they also exhibited wide variations in the other sources of variation. The average repeatability of these two regions were 45% and 75%, whereas for the other three regions it was about 99%. The FTIR spectrum of caprine milk could probably be used to predict several traits and to authenticate the origin of goat milk.

## 1. Introduction

Fourier-transform infrared spectroscopy (FTIR) is a high-throughput method with multiple applications that has revolutionized the livestock sector [1]. FTIR technology measures the vibrations of the atoms in a molecule related to their bond strengths. When the frequency of the IR radiation directed at the bond is equal to the frequency of the bond’s vibration, the bond absorbs the radiation. The frequencies absorbed constitute the molecule’s IR spectrum. Analyzing infrared spectra can tell us what molecules (hence, what compounds) are present in a sample (of milk, cheese, meat, etc.) and at what concentrations.

According to Smith, 2011 [2], infrared spectroscopy is almost universal, in the sense that the infrared spectra of solids, liquids, and gases can all be measured. A second advantage concerns the richness of information obtained: the position of a spectral peak reveals the structure of the molecules, the peak intensity reveals the concentration of molecules, and the peak width is sensitive to the chemical matrix. Further key features of FTIR are that it is relatively easy, fast, and sensitive, i.e., it is a non-destructive method that requires only grams/milliliters of material to produce a good spectrum. The main disadvantage of FTIR with regard to milk samples is the presence of water, which has intense peaks that can mask the spectra of the milk components.

In milk analyses, transmittance is defined as $T=I_{m}/I_{w}$, where $I_{m}$ and $I_{w}$ represent the transmitted radiation of milk and water (reference or identity testing), respectively. Usually, $I_{m}<I_{w}$ (and T<1) due to the presence of other milk components that affect the transmittance radiance, but it may also be that $I_{m}>I_{w}$ (and T>1). Another related metric is absorbance, defined as $A=-\log_{e}T$. Note that, by construction, the values of *T* are centered at one, so when taking the base e logarithm, *A* is centered at zero. When conducting quantitative analyses, absorbance is preferred because it is linearly proportional to concentration, according to Beer’s law [2].

FTIR spectrometry can be used—with different levels of accuracy—for:

i. the prediction of milk components than can be easily distinguished from each other due to the specific vibrational properties of their chemical bonds;
ii. the prediction of groups of milk components with similar chemical and vibrational properties;
iii. the prediction of the chemical components or physical-technological characteristics of milk that do not have specific vibrational properties;
iv. the prediction of the metabolic characteristics of animals affecting certain properties of milk;
v. the authentication of the origin of milk.

Fourier-transform infrared spectroscopy is, of course, most commonly used to predict milk components with specific vibrational properties with highly accurate results guaranteed. This is the case for the major chemical components of milk (fat, protein, and lactose), the predictions of which are regulated by the International Organization for Standardization (ISO) and certified by the International Committee of Animal Recording [3,4,5].

Although prediction of the total milk content of fat and protein is accurate, the quantification of individual milk fatty acids [6,7,8] or individual protein fractions [9,10,11] is much less accurate. In fact, the chemical and vibrational characteristics of the members of the same chemical family are very similar, so discriminating one milk fatty acid from another one is not simple, and the same is true when comparing a milk protein fraction with another one. In this case, prediction is based not only on the chemical bonds of each compound, but probably also on the relationships between the concentration of the compound in question and other characteristics of the milk [12].

Predicting chemical compounds without specific vibrational characteristics, such as minerals [13,14,15], and the physical-technological properties of milk [16,17,18] is based substantially on covariance between the compound or trait in question and others related in some way to it. Accuracy in such cases is never very high and depends on the closeness of association and the specificity of the vibrational properties of the associated compounds.

The structure of the covariance matrix is also fundamental to predicting traits defining the metabolism of animals using milk FTIR spectroscopy. Examples include the prediction of blood metabolites [19,20], animal dismetabolism [21], nutritional efficiency [22,23], animal energy balance [24,25], enteric methane emissions [26,27,28], fertility [29,30,31], etc.

Lastly, infrared spectra can be used to determine the fingerprint of milk for authentication purposes where adulteration is suspected [32,33,34], or to certify the area of origin [35], or the farming system in which the milk was produced [36,37]. Some other secondary method has been proposed for the rapid prediction of some substance or property of milk, but none has the versatility of FTIR spectrometry in that, with just one sample and one instrument, in a single passage, many components and characteristics of the milk, the dairy animal, and the dairy system can be predicted. Moreover, provided that the spectra are stored, new traits can be predicted a posteriori, simply using new calibration equations on old spectra.

Many studies have been carried out on milk from bovine species, but there has been very little research on the milk of other species, particularly goats [38,39,40].

The characteristics of bovine FTIR milk spectra have been extensively studied with the aim of understanding the properties of different fractions of the spectrum and of individual wavelengths, and in order to identify the areas related to specific chemical bonds. In a previous study on the FTIR spectrum of bovine milk, we clearly identified five major spectral fractions in the range of near-infrared (NIR), or short-wave infrared (SWIR), to mid-infrared (MIR, or MWIR) and long-infrared, or long-wave-infrared (LWIR) radiation [41]. Aside from phenotypic properties, bovine milk spectra have also been analyzed to identify the possible genetic parameters of wavelength absorbance [42,43,44] and to obtain genomic information [45,46].

Given that, unlike bovine milk, there is little knowledge of the FTIR spectrum of goat milk, and that this knowledge is needed for the correct use of goat spectra to predict and interpret chemical contents and metabolic properties, the aims of this research were as follows: (1) to study the absorbance values and their phenotypic variances of each wavenumber of the goat milk spectrum and to compare them with the characteristics of bovine milk spectrum from the literature; (2) to estimate the major components of phenotypic variance; and (3) to estimate the repeatability of 1060 wavenumbers in the FTIR region from 5000 to 930 × cm^−1^ of goat milk samples.

## 2. Materials and Methods

### 2.1. Experimental Design

This study is part of a research project (the Good-Milk project), which mainly aims to study the qualitative properties of goat milk compared to milk from other dairy species, with particular emphasis on milk protein fractions and genetic variants. The work package dedicated to the study and use of FTIR spectra to predict the qualitative traits of goat milk focuses on sampling milk from many goats representing different farming systems, breeds, parities, and lactation stages.

The 657 goats sampled for the present study were of 6 different breeds and were reared on 20 farms in Sardinia (Italy). The farms were classified into 3 dairy systems (traditional, intermediate, and modern) according to the feeding system, farm management, and conditions in the facilities. Information on the farming systems, animals, and sampling procedure are reported in a previous study [47]. Information on the qualitative and technological traits of the milk samples is provided in other studies carried out on the same database [48,49,50]. The goats belonged to the following breeds: Saanen (41 goats); Camosciata delle Alpi (164); Murciano-Granadina (143); Maltese (122); Sarda (44); and Sarda Primitiva (143). Parities on the day of sampling ranged from 1 to 15, and days in milk (DIM) ranged from 10 to 224.

A 50 mL milk sample was collected from each goat and stored immediately at 4 °C. Within 24 h of sampling, FTIR spectra (2 replicates per milk sample) were obtained with a MilkoScan FT6000 milk analyzer (Foss A/S, Hillerød, Denmark). Absorbance values of 1060 spectral wavenumbers from 5000 to 930 × cm^−1^ were recorded. Parity was recoded from a count variable to a factor with six levels (1–2, 3, 4, 5, 6, 7+), while DIM was recoded from a count variable to a factor with five levels (1–60, 61–90, 91–120, 121–150, 151–240).

### 2.2. Statistical Model

A database of 1,392,840 absorbance values (657 samples/goats × 2 replicates × 1060 wavenumbers) was compiled. Outlier spectra were checked on the basis of the Mahalanobis distance. A linear mixed model was fitted to estimate the variance components and repeatability of the milk absorbance at each individual FTIR wavenumber. The model was as follows:

(1) $$y_{ijklm}=\mu +G_{i}+B_{j}+F_{k}+P_{l}+D_{m}+e_{ijklm},$$

where $y_{ijklm}$ represents the 1314 absorbance values (A) recorded for a particular wavenumber (657 samples/goats in duplicate), *μ* is the overall absorbance mean or intercept (fixed) for a particular wavenumber, $G_{i}\sim iid\;N\left(0,\sigma_{G}^{2}\right)$ is the random effect of the ith sample/goat, $B_{j}∼iid\;N\left(0,\sigma_{B}^{2}\right)$ is the random effect of the jth breed, $F_{k}\sim iid\;N\left(0,\sigma_{F}^{2}\right)$ is the random effect of the kth flock, $P_{l}\sim iid\;N\left(0,\sigma_{P}^{2}\right)$ is the random effect of the lth parity, $D_{m}\sim iid\;N\left(0,\sigma_{D}^{2}\right)$ is the random effect of the mth days in milk, and $e_{ijklm}\sim iid\;N\left(0,\sigma_{e}^{2}\right)$ is the model residual. Here, N(⋅,⋅) stands for a normally distributed random variable, and ‘iid’ stands for one that is independent and identically distributed. Strictly speaking, model (1) is a linear mixed model where μ is the only fixed effect and the other components are treated as random. All random components were assumed to be independent of each other. This model was fitted 1060 times (one per wavenumber) using the BGLR-R package [51] in the R programming language [52].

From the 1060 individual mixed models as per Equation (1) fitted to each wavenumber of the FTIR spectrum, the variance components of the random effects, i.e., ${\hat{\sigma }}_{G}^{2},\;{\hat{\sigma }}_{B}^{2},\;{\hat{\sigma }}_{F}^{2},\;{\hat{\sigma }}_{P}^{2},{\hat{\sigma }}_{D}^{2}$, the residual term ${\hat{\sigma }}_{e}^{2}$, and the computed proportion of variance explained by each term were estimated. Under additivity, the estimate of total (phenotypic) variance is calculated as the sum of the variance components, ${\hat{\sigma }}_{Ph}^{2}={\hat{\sigma }}_{G}^{2}+{\hat{\sigma }}_{B}^{2}+{\hat{\sigma }}_{F}^{2}+{\hat{\sigma }}_{P}^{2}+{\hat{\sigma }}_{D}^{2}+{\hat{\sigma }}_{e}^{2}$, and sample repeatability is expressed as $R_{sample}=\frac{{\hat{\sigma }}_{G}^{2}+{\hat{\sigma }}_{B}^{2}+{\hat{\sigma }}_{F}^{2}+{\hat{\sigma }}_{P}^{2}+{\hat{\sigma }}_{D}^{2}}{{\hat{\sigma }}_{Ph}^{2}}$. Sample repeatability was calculated as the sum of the variances due to the random effects included in the model as a percentage of the phenotypic variance (i.e., the sum of the variance of the random factors plus the residual variance).

## 3. Results

### 3.1. Descriptive Statistics of the Goat Milk Spectra

**Figure 1:**
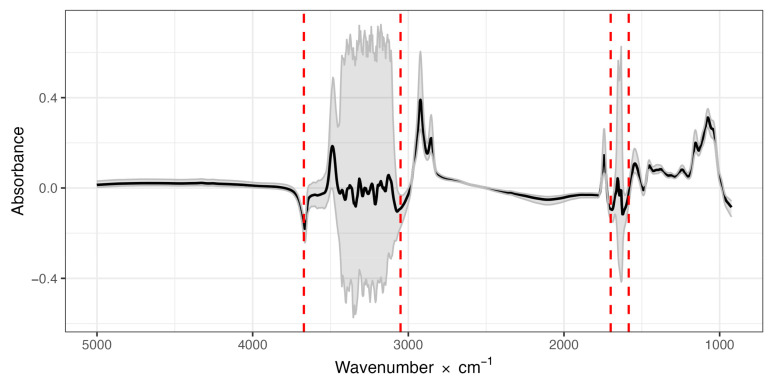
Mean absorbance values (dark solid line) and the 0.025 and 0.975 quantiles (gray lines) of the 1060 individual infrared wavelengths (5000 to 930 × cm^−1^), measured from 1314 spectra of milk samples from 657 goats. Red dashed lines indicate regions with high variability.

Figure 1 depicts the mean phenotypic absorbance values and the 0.025 and 0.975 quantiles of the milk samples for the entire FTIR spectrum (1060 individual wavelengths) obtained from the milk of the 657 goats included in the study (1314 milk spectra). The two regions with very high phenotypic variability in absorbance values in the goat milk dataset, which are characterized by the wavelengths approximately between 3669 and 3052 and between 1698 and 1586, are of note. These regions are known to be water zones in cow milk samples and are indicated by peaks of water absorption that can mask the effects of other milk components. Goat milk spectra are therefore similar to cow milk spectra, and we will refer to these regions henceforth as the water zones or water regions.

### 3.2. Phenotypic Analysis of the FTIR Spectra

**Figure 2:**
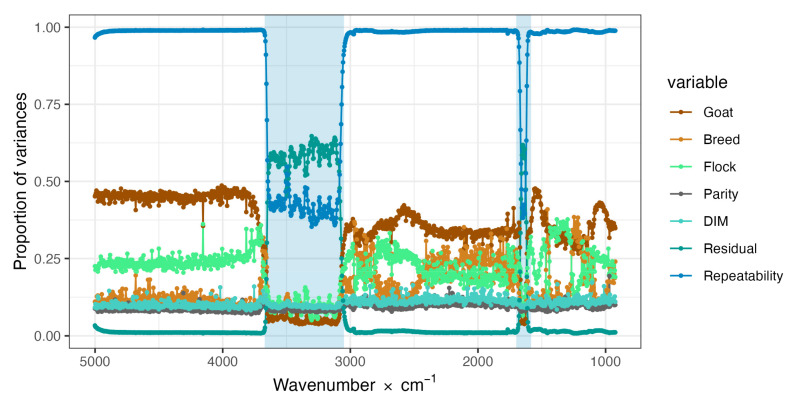
Sample/goat repeatability and proportions of variances for Goat, Breed, Flock, Parity, Days in Milk (DIM), and the residual term for the absorbance values of each of the 1060 wavenumbers analyzed.

Figure 2 shows the sample/goat repeatability for the 1060 analyses carried out on the entire goat milk spectrum, i.e., the infrared region between wavelengths 2.0μm (wavenumber 5000 × cm^−1^) and 10.8μm (wavenumber 930 × cm^−1^), and the percentage variance explained by each random effect included in model (1) (Goat = G, Flock = F, Herd = H, Parity = P, and Days in milk = D) and the residual. As can be seen, the sample/goat repeatability approaches one for almost the entire spectrum, with the exception of the two regions with very high variability (Figure 1), where it is significantly lower because the residual variance often increases to values of 50% of phenotypic variance. In the other regions, the sample/goat random effect explains an average of 39% of the phenotypic variance, versus 9% in the aforementioned two regions. The random effect of flock explained, on average, 23% in the group of regions with low variability, and 11.8% in the group with high variability, while the random effect of breed explained, on average, 17% and 11%, respectively, of phenotypic variability. There was little variation in the contributions of the random effects of Parity (9%) and Days in Milk (11%) to the phenotypic variability in the entire spectrum. Finally, the unexplained phenotypic variation (residual) was an average of 1% in the low and 50% in the high variability regions of the spectrum.

## 4. Discussion

The discussion deals separately with the three specific aims of this study as follows: the patterns and phenotypic variances in the absorbance values of the goat milk spectrum; the estimation of the variance components of the major sources of variation of infrared absorbance; and the repeatability of 1060 wavenumbers in the FTIR spectrum of goat milk samples.

### 4.1. The Patterns and Phenotypic Variances in the Absorbance Values of the Goat Milk Spectrum

The infrared region analyzed in this study (wavelengths 2.0 to 10.8 μm, or wavenumbers 5000 to 930 × cm^−1^) is a section of the near-, mid-, and long-infrared regions of the electromagnetic spectrum. The spectrometer used in this study is the one most commonly used to predict the composition of milk samples [53] in many countries of the world, especially within milk recording systems for the genetic improvement of dairy populations. It is widely used to analyze not just cow milk samples, but also buffalo [33], sheep [39,54,55], and goat [34,39,56] milk samples with specific calibrations [3,56].

Given that milk spectra can be expressed in different ways (as transmittance or absorbance, as the entire spectrum or specific regions, etc.), it is worth noting that the average spectrum obtained here for goat milk is very similar to that frequently obtained for cow milk.

In our previous study on the variability of FTIR spectra of bovine milk samples [41], we analyzed milk in the same wavenumber interval using the same type of spectrometer, but the spectra were expressed as transmittance, not absorbance, values. This explains why the spectra are centered on zero in this study, whereas in the previous study they were centered on a value of one, and why the pattern is reversed in the sign with respect to the center. Other studies obtained very similar patterns with transmittance spectra [43]. The absorbances and patterns reported for bovine milk in a subsequent study [57] were very similar to those observed here for caprine milk (Figure 1). Other studies on the absorbance FTIR spectra of bovine milk have also reported a similar pattern to that of caprine milk found here [58].

The phenotypic variation of absorbance, shown in Figure 1, was very different at every wavenumber analyzed. The two spectral regions with a much greater variation in absorbance than the rest of the spectrum were identified as the areas of absorbance of the O-H chemical bond, and therefore highly influenced by the presence of water. A large proportion of milk is constituted by water, so the transmittance (and consequently, the absorbance) spectrum of milk is very similar to that of water [59]. The milk spectrum is, as in the present study, frequently expressed as the ratio between the values measured in milk and those measured in pure water, taken as a reference. Based on the average values and standard deviations observed along the milk spectrum, and taking into account the heritability coefficients estimated for every wavenumber measured, in our previous study we proposed subdividing the spectrum of bovine milk into five sections [41]. Given the close similarity observed here, we decided to use the same subdivisions for goat milk, represented by the vertical dashed red lines in Figure 1 and the different background color of the areas in Figure 2 (white or light blue). The first (SWIR-MWIR) of the two “water” spectral regions is identified in the area of transition between the near- and mid-infrared (NIR and MIR) radiations (wavelength 2.73 to 3.27 μm) and the second (MWIR2) region in the central area of mid-infrared radiation (5.89 to 6.31 μm).

The two “water” spectral regions are often excluded when milk spectra are used to predict milk traits with appropriate chemometric procedures, as they are considered sources of “noise” and inexplicable variations [42]. However, the O-H bond is also present in many other chemical compounds that are important for defining milk quality, while other chemical bonds have been shown to correspond to the absorbance of electromagnetic radiation in these sections, and, lastly, the absorbance of several wavelengths in these sections has been found to be, in part, genetically controlled [43,46].

A better understanding and discussion of the role and importance of different spectral regions of the goat milk spectrum could be had by quantifying the major sources of variation in milk absorbance.

### 4.2. Variance Components of the Major Sources of Variation of Infrared Absorbance

In light of the results (see Figure 2), the phenotypic variability (${\hat{\sigma }}_{Ph}^{2}$) of milk absorbance at each wavenumber was divided into their major sources of variation, and the variability due to individual sample/goat (${\hat{\sigma }}_{G}^{2}$), breed of goat (${\hat{\sigma }}_{B}^{2}$), flock (${\hat{\sigma }}_{F}^{2}$), parity (${\hat{\sigma }}_{P}^{2}$), stage of lactation (${\hat{\sigma }}_{D}^{2}$), and the residual variation (${\hat{\sigma }}_{e}^{2}$), and computed the sample/goat repeatability was treated as random effects. Bear in mind that in this study, only one milk sample (with two spectral replicates) per goat was taken, so the effects of individual goat and sample are combined, whereas the residual variation expresses the differences between the two spectral replicates obtained from each milk sample.

To facilitate discussion, these estimates were averaged according to the five spectral regions proposed in our previous study; these are summarized in Table 1.

The first of these five regions is the near-infrared or short-wavelength region (SWIR, 2–2.72 μm), followed by the first “water” region (SWIR-MWIR, 2.73–3.27 μm), the mid-infrared 1 region (MWIR-1, 3.28–5.88 μm), the second “water” region (MWIR-2, 5.89–6.31 μm), and lastly, the mid- to long-infrared (MWIR-LWIR, 6.32–10.76 μm) region. To facilitate comparison with other studies, equivalences in standard ISO, wavenumber (cycles per inverse centimeter, waves × cm^−1^ and frequencies (cycles per second, Hertz) are also listed in Table 1.

It is worth noting that the average absorbance of the wavenumbers in the two “water” regions is negative, whereas it is positive in the other three spectral regions. Moreover, almost one third of the wavenumbers had an average absorbance of less than 1 standard deviation from the overall mean. At the same time, in these two regions, 19% and 7% of the wavelengths had an average absorbance greater than 1 standard deviation from the overall mean, and the average phenotypic variability was 6 to 22 times larger than in the other three spectral regions (Table 1). Regarding the variance components, it is clear from the same table that these large differences in phenotypic variability are reflected in the variability in all the major sources of variation. It is therefore expected that, for each variance component, the “water” regions will exhibit the largest proportion of wavenumbers characterized by very high variance, while the other three regions will be characterized by very low variance.

As the absorbance values measured at each wavenumber are centered and standardized before being used to predict milk traits, it is of interest to analyze the relative importance of different sources of variation in the five spectral regions. The sample/goat variance, expressed as an average of all the wavenumbers in the entire FTIR caprine milk spectrum (Table 1), accounts for one third of the phenotypic variance, and is, on average, greater in the three “non-water” regions and lower in the “water regions” (7% in the SWIR-MWIR and 22% in the MWIR2). It is worth noting that there were smaller differences in the effects of breed of goat among the five spectral regions, with average proportions ranging from 10% to 21%, and the effects of flock were similar in importance and variability, with values ranging from 10% to 25%. The individual goat factors that change with time (parity and stage of lactation) had a smaller, but significant, influence on infrared radiation absorbance, and presented similar values in the five spectral regions (8% to 10% for the effect of parity, 10% to 12% for stage of lactation).

No studies in the literature that analyze the major sources of variation in the absorbance of electromagnetic radiation at the level of individual wavenumbers or spectral regions for milk was found, but there are some studies, described later, that quantified the genetic and environmental components of the phenotype. As seen here, both components are much greater in the regions with a very large phenotypic variability (“water” regions) than in the rest of the spectrum [41]. In this case, too, it might be more informative to analyze the relative proportions of the genetic and environmental variance components, which are expressed as the heritability of absorbance. It is worth noting that our previous studies, among others, found heritabilities, albeit variable, for several wavenumbers also in the “water” regions [42,44]. Obviously, the genetic variance represents the major part of the variation due to the breed and a part of that due to the individual animal. The herd–year–season component has also been found to have a strong influence on the phenotypic variance of absorbance in bovine milk [44], and to affect the prediction of milk traits from FTIR caprine milk spectra [60]. Interactions between the cow’s genetics, parity, and stage of lactation and spectral region have also been found [44].

We can conclude that the two “water” regions are affected by individual sample/goat, breed, herd, parity, and stage of lactation, albeit to a lesser extent than the other regions, and that they therefore probably contain valuable information that could be used in the prediction of milk traits or the authentication of breed and feeding and production systems, provided that it is combined with suitable chemometric methods.

It is worth noting that breed of cow had very little effect on the patterns and variability in the FTIR spectra of bovine milk [61]; however, when calibration equations developed on one breed for predicting milk traits were then applied to other breeds, validation accuracy tended to be slightly lower than when the calibration equations were developed on multi-breed training sets. Similar results were obtained in a study predicting milk coagulation traits in four goat breeds [62]. As the spectra were not compared in either of the two studies, it is unclear whether the different results with different breeds are due to inherent differences in the predictors (FTIR spectra) or to the different values and characteristics of the predicted traits.

### 4.3. Animal/Sample Repeatability of the Absorbance of 1060 Wavelengths of FTIR Goat Milk Spectra

The variability not captured by the random effects included in the model is captured by ${\hat{\sigma }}_{e}^{2}$. As can be seen from Figure 2, the relative contribution of the error term to the total phenotypic variance of absorbance is close to zero for the SWIR, MWIR-1, and MWIR-LWIR regions, while for the two “water” regions (SWIR-MWIR and MWIR2), it rose dramatically to more than 50%. In fact, the average proportion of phenotypic variance not explained by the random term is 10% over the entire spectrum, but in the SWIR, MWIR-1, and MWIR-LWIR regions, it is 1.1%, 1.3%, and 1.4%, respectively, whereas in the “water” regions (SWIR-MWIR and MWIR-2), it is 55% and 25%, respectively. Conversely, as defined in this study, the repeatability of the absorbance measures is the complement of the residual proportion of phenotypic variance and is almost 99% in the three “non-water” spectral regions and about 45% and 75% in the two “water” regions. Given the different structures of the sources of variation of the absorbance spectrum, when using the entire spectrum (including the two “water” regions), it seems advisable to use the average of two–three replicates per milk sample instead of a single spectrum, or to use chemometric procedures that can select the most informative wavenumbers.

There are very few if any data in the literature regarding the repeatability of FTIR absorbance measured at each wavenumber. In the case of bovine meat, we also found large differences in repeatability along the NIR spectrum [63]: it was highly variable in the region of the electromagnetic spectrum corresponding to ultraviolet and visible light (0.35 to 0.74 μm), relatively high (about 80%) for wavenumbers in the IR-A region (NIR: 0.74 to 1.40 μm), and very low (10% to 30%) in the IR-B interval (SWIR: 1.40 to 1.85 μm). The heritability of the individual wavelengths were, correspondingly, very different [64].

## 5. Conclusions

Our study on a representative goat population (different farming systems, breeds, parities, and lactation stages) shows that the FTIR spectrum of caprine milk has many similarities with that of bovine milk reported in the literature. The major sources of variation were as follows: sample/goat (33% of the total); flock (21%); goat breed (15%); lactation stage (11%); parity (9%); and the residual unexplained variation (10%). As in cattle species, the spectrum is highly heterogeneous, and it was possible to distinguish five regions, two of which (“water” regions) presented much larger variability than the others, not only in terms of the residual variation, but also in terms of the effects of the major sources of variation. The similarity with the bovine milk spectrum, as well as the high repeatability (90% for the entire spectrum, 99% in the non-water regions), leads us to expect that caprine milk spectra could also be a valuable tool for predicting many milk properties.

## Figures and Tables

**Table 1:** Characteristics of the five regions of the FTIR spectrum of goat milk.

Item ^2^	Entire Spectrum	SWIR ^1^	SWIR-MWIR ^1^	MWIR-1 ^1^	MWIR-2 ^1^	MWIR-LWIR ^1^
ISO	NIR-MIR	NIR	NIR-MIR	MIR	MIR	MIR
Wavenumber, ${\mathrm{cm}}^{-1}$	5000–930	5000–3673	3669–3052	3048–1701	1698–1586	1582–930
Wavelength, μm	2.00–10.76	2.00–2.72	2.73–3.27	3.28–5.88	5.89–6.31	6.32–10.76
Frequency, THz	149.9–27.9	149.9–110.1	110.0–91.5	91.4–51.0	50.9–47.5	47.4–27.9
Waves tested, no.	1060	347	161	350	31	171
Absorbance:	medium	medium	low	medium	low	high
Average absorbance	0.0186	0.0109	−0.0109	0.0109	−0.0506	0.0910
Waves >0.130 ^a^, %	10	0	19	10	7	25
Waves <−0.093 ^b^, %	7	2	31	1	31	1
Phenotypic variability:	medium	very low	very high	low	high	low
Mean of ${\hat{\sigma }}_{Ph}$	0.043	0.008	0.206	0.011	0.105	0.015
Waves ${\hat{\sigma }}_{Ph}>0.09\;$^c^, %	12	0	69	1	36	0
Waves ${\hat{\sigma }}_{Ph}<$0.02 ^d^, %	74	99	0	86	4	77
Animal (Goat) variability:						
Mean of ${\hat{\sigma }}_{G}$	0.013	0.006	0.046	0.007	0.031	0.009
Proportion of ${\hat{\sigma }}_{Ph}^{2}$	0.33	0.45	0.07	0.35	0.22	0.36
Breed variability:						
Mean of ${\hat{\sigma }}_{B}$	0.014	0.003	0.063	0.005	0.034	0.006
Proportion of ${\hat{\sigma }}_{Ph}^{2}$	0.15	0.12	0.10	0.21	0.13	0.18
Flock variability:						
Mean of ${\hat{\sigma }}_{F}$	0.015	0.004	0.063	0.005	0.038	0.007
Proportion of ${\hat{\sigma }}_{Ph}^{2}$	0.21	0.24	0.10	0.22	0.20	0.25
Parity variability:						
Mean of ${\hat{\sigma }}_{P}$	0.013	0.002	0.059	0.004	0.031	0.005
Proportion of ${\hat{\sigma }}_{Ph}^{2}$	0.09	0.09	0.08	0.10	0.09	0.10
Lactation stage variability:						
Mean of ${\hat{\sigma }}_{D}$	0.014	0.003	0.064	0.004	0.033	0.005
Proportion of ${\hat{\sigma }}_{Ph}^{2}$	0.11	0.10	0.10	0.12	0.10	0.11
Repeatability:						
Mean Repeatibility	0.90	0.99	0.45	0.99	0.75	0.99

^1^: SWIR = short-wave infrared; MWIR = mid-wave infrared; LWIR = long-wave infrared; ^2^: ${\hat{\sigma }}_{\mathrm{Ph}}^{2}$ is the phenotypic variance calculated as the sum of the variance components of the random effects included in model 1 (goat, breed, flock, parity, lactation stage, and residual) [${\hat{\sigma }}_{\mathrm{Ph}}^{2}={\hat{\sigma }}_{G}^{2}+{\hat{\sigma }}_{B}^{2}+{\hat{\sigma }}_{F}^{2}+{\hat{\sigma }}_{P}^{2}+{\hat{\sigma }}_{D}^{2}+{\hat{\sigma }}_{e}^{2}$ ]; the mean of ${\hat{\sigma }}_{\;i}^{}$ is the mean of the standard deviation for the ith random effect of model 1 for the waves tested in the whole spectrum and in each of the five regions; the proportion of ${\hat{\sigma }}_{\mathrm{Ph}}^{2}$ is the proportion of the phenotypic variance explained by the ith random effect of the model 1 [${\hat{\sigma }}_{i}^{2}/{\hat{\sigma }}_{\mathrm{Ph}}^{2}$ ]; repeatability is the proportion of the sum of the variances explained by the random effects (without the residual one) on the ${\hat{\sigma }}_{\mathrm{Ph}}^{2}$ [${\;R}_{\mathrm{sample}}=\frac{{\hat{\sigma }}_{G}^{2}+{\hat{\sigma }}_{B}^{2}+{\hat{\sigma }}_{F}^{2}+{\hat{\sigma }}_{P}^{2}+{\hat{\sigma }}_{D}^{2}}{{\hat{\sigma }}_{\mathrm{Ph}}^{2}}\;]$; ^a^: Proportion of waves in the region (% of the total waves in the region) with a value higher than twice the average of the entire spectrum; ^b^: number of waves in the region (% of the total waves in the region) with a value higher than half the average of the whole spectrum; ^c^: number of waves in the region (% of the total waves in the region) with a value higher than the average + 1 SD of the entire spectrum; ^d^: number of waves in the region (% of the total waves in the region) with a value lower than the average − 1 SD of the entire spectrum.

## Data Availability

Data can be obtained from the authors.
